# No Effects of Stimulating the Left Ventrolateral Prefrontal Cortex with tDCS on Verbal Working Memory Updating

**DOI:** 10.3389/fnins.2017.00738

**Published:** 2018-01-10

**Authors:** Karolina M. Lukasik, Minna Lehtonen, Juha Salmi, Marcus Meinzer, Juho Joutsa, Matti Laine

**Affiliations:** ^1^Department of Psychology, Abo Akademi University, Turku, Finland; ^2^Department of Psychology and Logopedics, Faculty of Medicine, University of Helsinki, Helsinki, Finland; ^3^Department of Psychology, University of Turku, Turku, Finland; ^4^Centre for Clinical Research, The University of Queensland, Brisbane, QLD, Australia; ^5^Athinoula A. Martinos Center for Biomedical Imaging, Massachusetts General Hospital and Harvard Medical School, Charlestown, MA, United States; ^6^Berenson-Allen Center for Noninvasive Brain Stimulation, Beth Israel Deaconess Medical Center and Harvard Medical School, Boston, MA, United States; ^7^Department of Neurology, University of Turku, Turku, Finland; ^8^Turku Brain and Mind Center, University of Turku, Turku, Finland

**Keywords:** brain stimulation, n-back, tDCS, ventrolateral prefrontal cortex, verbal working memory

## Abstract

The effects of transcranial direct current stimulation (tDCS) on dorsolateral prefrontal cortex functions, such as working memory (WM), have been examined in a number of studies. However, much less is known about the behavioral effects of tDCS over other important WM-related brain regions, such as the ventrolateral prefrontal cortex (VLPFC). In a counterbalanced within-subjects design with 33 young healthy participants, we examined whether online and offline single-session tDCS over VLPFC affects WM updating performance as measured by a digit 3-back task. We compared three conditions: anodal, cathodal and sham. We observed no significant tDCS effects on participants' accuracy or reaction times during or after the stimulation. Neither did we find any differences between anodal and cathodal stimulation. Largely similar results were obtained when comparing subgroups of high- and low-performing participants. Possible reasons for the lack of effects, including individual differences in responsiveness to tDCS, features of montage, task and sample characteristics, and the role of VLPFC in WM, are discussed.

## Introduction

Working memory (WM) is a multi-component system involved in temporary maintenance and updating of information (Baddeley, 2002), enabling a dynamic and purposeful interaction with the environment. WM differs notably from other memory systems with regard to its restricted capacity and the temporal decay of stored information, although the exact capacity limits and the way information is maintained in WM are still debated (Gobet and Clarkson, 2004; Cowan, 2010). Due to these constraints, information stored in WM needs to be constantly maintained and updated, which entails active rehearsal of relevant information, inhibition of irrelevant stimuli, and encoding of new representations for WM storage. Maintenance is thought to be more automatic, whereas updating requires monitoring, selection and active control so that new relevant stimuli can enter the WM storage (Morris and Jones, 1990; Kessler and Oberauer, 2015). As updating appears to be cognitively more costly than maintenance (Kessler and Meiran, 2008) and thus potentially more sensitive to the facilitatory effects of tDCS, we selected a WM updating task for the present study.

Due to its relative ease of use and non-invasive character, transcranial direct current stimulation (tDCS) has become a popular technique in studies investigating cognitive enhancement (Berryhill and Jones, 2012; Martin et al., 2013; Au et al., 2016). However, there is heterogeneity even in the results of motor studies (Wiethoff et al., 2014), and the inherent complexity of cognitive tasks may make it even more challenging to determine the effects of tDCS on cognition (Jacobson et al., 2012). However, recent research has shed light on the sources of variability in brain stimulation experiments. Nixon et al. (2004) report that effectiveness of TMS depended on both timing and precise localization. In turn, studies on tDCS and visuospatial WM have shown that response to stimulation may vary depending on cognitive load (Wu et al., 2014) and initial performance levels, so that low-performing participants would benefit from tDCS while high-performers would not (Tseng et al., 2012; Hsu et al., 2014, 2016; Wu et al., 2016; Juan et al., 2017). As Krause and Kadosh (2014) point out, the effectiveness of stimulation depends on initial brain state, and baseline performance is a crude yet valuable indicator of it. Furthermore, different electrode montages affecting specific brain networks interact with ongoing neural processes and tDCS effects also depend on the specifics of the experimental design (Fertonani and Miniussi, 2017).

Working memory (WM) is one of the cognitive functions most frequently investigated using tDCS, but little is known about how stimulation over the specific brain regions thought to be associated with specific WM subprocesses affects task performance. To date, the most frequently studied region has been the dorsolateral prefrontal cortex (Brunoni and Vanderhasselt, 2014; Wu et al., 2014; Hill et al., 2016; DLPFC). In the present study we chose to stimulate ventrolateral prefrontal cortex (VLPFC), since brain imaging studies have shown that it plays a key role in WM (Veltman et al., 2003; Owen et al., 2005) as well as in several other cognitive functions (Ridderinkhof et al., 2004). To our best knowledge, effects of applying tDCS to VLPFC on WM performance have not been studied before.

We employed a complex WM updating task, an n-back task, which requires the participant to monitor a constantly changing series of stimuli and to decide whether the current stimulus is the same as the one presented *n* trials back. This task allows manipulation of WM load by changing the value of *n*, which is reflected in monotonic decrease in accuracy and increase in reaction times (RTs) as *n* is increased (Jonides et al., 1997; Jaeggi et al., 2010). As the n-back task taps into many subprocesses involved in WM (see Schmiedek et al., 2014), it has been a popular tool in neuroimaging studies to examine WM load and modality effects on brain activations. A meta-analysis of n-back neuroimaging studies by Owen et al. (2005) revealed the involvement of large-scale fronto-parietal networks, with some modulation of activity patterns by specific n-back task characteristics. For example, dorsolateral prefrontal cortex (DLPFC) activity during WM has been suggested to reflect strategic control of WM and reorganizing the stimuli into structures (“chunks”). Studies using repetitive transcranial magnetic stimulation (rTMS) have given further evidence for DLPFC involvement in WM, showing that applying rTMS to this region impairs WM performance (Mottaghy et al., 2000; Sandrini et al., 2008). Narayanan et al. (2005) showed that DLPFC has more connectivity with parietal cortex than VLPFC, which points to the role of dorsolateral regions in coordinating WM networks. On the other hand, the VLPFC has been linked to explicit retrieval, response sequencing and inner speech (Owen et al., 2005) and also to subvocal phonological rehearsal required in verbal WM tasks (Crottaz-Herbette et al., 2004). In addition to the rehearsal function, this region has also been related to verbal WM updating (Veltman et al., 2003; Owen et al., 2005), stimulus selection, and resolving interference which is crucial for updating the contents of WM storage (Nee et al., 2007). As the persistent activation of VLPFC in WM tasks is interpreted as evidence for the rehearsal mechanism (Crottaz-Herbette et al., 2004) and VLPFC is also assumed to contribute to other WM subprocesses (Goulden et al., 2014), stimulating this region can be expected to enhance WM performance. In spite of well-established neuroimaging evidence of activation of the left VLPFC in verbal WM tasks (Manelis and Reder, 2014), this area has not been targeted in previous tDCS studies of WM. TDCS studies focusing on other cognitive functions have found that the left VLPFC stimulation facilitates speech production and phonemic and semantic word fluency (Cattaneo et al., 2011; Meinzer et al., 2012) as well as picture naming (Sparing et al., 2008). Its role in both WM and language tasks makes the left VLPFC thus an interesting potential stimulation target in studies of verbal WM.

The idea that WM performance could be influenced by tDCS stems directly from its working principle, namely inducement of transient changes in cortical excitability (Nitsche and Paulus, 2000). According to Stagg and Nitsche (2011), tDCS either induces a shift in resting membrane potential or modulates synaptic activity, resulting in hypopolarization of the neurons located near the anode and hyperpolarization of the neurons near the cathode. In other words, anodal stimulation is assumed to have an excitatory effect, while cathodal stimulation promotes inhibition and reduces neural firing. For example, a study by Zaehle et al. (2011) revealed that anodal stimulation of DLPFC enhanced performance on a WM task and amplified oscillatory power in theta and alpha bands, which also increased along with a greater WM load (Jensen and Tesche, 2002; Sauseng et al., 2005). Cathodal stimulation, in turn, impaired performance and reduced oscillatory power. Maintenance of information in WM is considered to rely on recurrent loops which uphold persistent activity levels in the prefrontal neurons (Durstewitz et al., 2000). Stimulating the neurons involved in WM may thus change the cortical excitability in such a way that facilitates the communication between regions in the recurrent loops.

In the light of previous findings, we chose to investigate whether applying tDCS to the left VLPFC would affect verbal WM updating performance in a digit 3-back task. We decided to maintain a steady, high WM load throughout the experiment, and the 3-back level was expected to provide a suitable level of difficulty with the present sample of participants. To the best of our knowledge, this is the first study examining the WM effects of tDCS over this brain region. We compared anodal and cathodal stimulation as well as sham, hypothesizing that anodal tDCS would enhance WM while cathodal tDCS would have a disruptive effect on performance compared to a placebo stimulation condition (i.e., sham tDCS). As some studies report improvement of WM not during stimulation but afterwards (see for example Ohn et al., 2008; Teo et al., 2011), we measured participants' WM performance both while receiving stimulation (“online”) and for 10 min following the stimulation (“offline”). We used a within-groups double-blind design where each participant underwent anodal stimulation, cathodal stimulation, and sham stimulation in a counterbalanced design. Due to the reported high individual variability in baseline task performance and training effects in the n-back task (Jaeggi et al., 2010), we also conducted an analysis that compared stimulation effects in high- vs. low-performing participants. As we used a within-subjects design and no prior evidence on the effects of tDCS to the left VLPFC during WM updating was available, we decided to use median split for the variable of interest (digit n-back performance) as a labeling factor. Moreover, as previous studies found that baseline performance levels predict stimulation response to tDCS (Peña-Gómez et al., 2011; Berryhill and Jones, 2012; Meinzer et al., 2013; Kim et al., 2014; Martin et al., 2017), we also examined the effects of stimulation at individual level by a gain-score analysis which takes into account the participant's baseline performance level before stimulation.

## Methods

### Participants

We recruited 34 healthy, right-handed young adults by e-mailing student associations and student councils in Turku and Helsinki. As one participant resigned from the study due to intense headache possibly caused by tDCS, the final sample included 33 participants (23 females, 10 males; age range 19–28 years, mean age 22.6 years, *SD* = 2.42). All participants were university students or graduates, including students from universities of applied sciences. The recruitment message contained a brief description of the study, information on the reimbursement, and a medical screening questionnaire. Exclusion criteria were left-handedness, neurological or psychiatric disorders, somatic conditions that might be relevant for the experiments, presence of medical devices (surgical clips, cochlear implants, drug pumps, etc.) in the body, history of traumatic brain injury, medication affecting the central nervous system, chronic headache, dizziness or vertigo, experience of seizures or an epileptic attack, family history of epilepsy, having undergone brain or vertebral column surgery, history of drug abuse, drug use in the last 4 months, diagnosed learning difficulties (dyslexia, dyscalculia, specific language impairment, etc.), large tattoos on the scalp or piercings in the head that cannot be removed, and pregnancy. Inclusion and exclusion criteria were evaluated using a questionnaire and interview. The final decision about the enrollment of the subjects was made by a licensed physician (JJ).

Prior to the study, each participant completed a background information questionnaire including questions about age, gender, and language use. Upon completing all study sessions, the participants were debriefed and received 45€ as reimbursement.

### Research design

**Figure 1:**
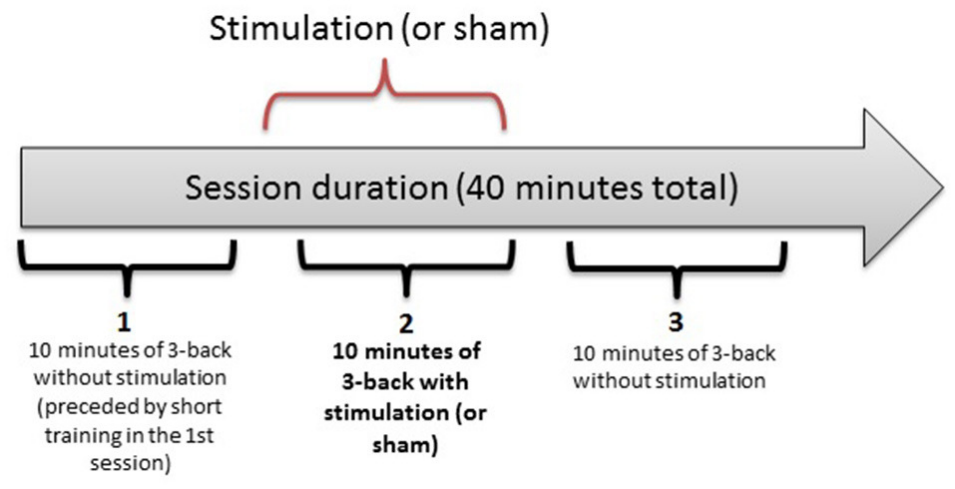
The three-block structure of each session in the experiment. Each 10-min bracket represents one block: 1 (baseline), 2 (online), 3 (offline).

In a double-blind, counterbalanced within-subjects design, each participant completed 3 separate sessions, receiving anodal, cathodal and sham stimulation. The sessions were performed on separate days, with a washout period of 48 h or more between sessions. For each participant, the sessions were held at the same time of the day (± 2 h) to minimize the influence of confounding variables on task performance. The first session was preceded by a short training block. Each session consisted of three 10-min n-back task blocks, as illustrated in Figure 1. After each block, there was a self-timed pause that was never longer than 5 min. After the last block of a session, the participants completed two questionnaires on side effects experienced during the study and possible long-term effects (in sessions 2 and 3). They also assessed the likelihood of receiving active stimulation or sham on a given day.

### Brain stimulation

We used the EMS BrainStim device with rubber electrodes. The electrode size was 25 cm2. Electrodes were placed in sponge pockets (anode pocket: 6 × 8 cm; reference electrode pocket: 6 × 5 cm) soaked in saline solution. The anode size was chosen to cover the entire left inferior frontal gyrus (IFG; F7 according to the 10–20 system). The anode was placed parallel to the IFG, with the shorter edge angled 45° relative to the supraorbital ridge. The reference electrode was placed over the right supraorbital cortex, its longer edge aligned with the supraorbital ridge.

The stimulation was double-blind, i.e., one researcher programmed the device prior to the session, and another one conducted the session with the participant. The screen of the device showed no information about stimulation type.

Both anodal and cathodal stimulation were administered with a constant current (1.5 mA) for 10 min. At the beginning and end of the experiment, the current was ramped up and down over 40 sec. In the sham condition, the participants experienced a 40-s electric current of 1.5 mA, also ramped up and down, at the beginning and at the end of the 10-min interval.

### The N-back task

In the present computerized visual-numerical n-back task programmed with the Presentation (Neurobehavioral Systems 2003–2016) software, the participants saw digits on the screen, one at a time, and they were to decide whether the digit was the same as the one shown 3 trials back. The task requires both maintenance and updating of information in WM. A 3-back task was chosen for its relative difficulty that should leave room for a possible stimulation-related improvement in reaction time (RT) and/or accuracy.

Three participants were excluded from the analysis because overall n-back accuracy rates across the three sessions were below chance level (cutoff at 58% correct on both targets and non-targets). The RTs (correct responses only) as well as the accuracy rates measured as d-prime values were analyzed by mixed-model ANOVAs with Stimulation type (anodal, cathodal, sham), Block (3 levels: first, second, third) and Session order (a dummy variable with 3 levels of counterbalancing order) as factors. For further analyses, we investigated individual gain scores, and compared subgroups of low- and high-performers based on median split.

### Modeling of current flow

We performed post-hoc modeling of electric current flow distribution for our montage using the COMETS2 software (Lee et al., 2016). COMETS2 is a MATLAB toolbox that uses a finite element method and a realistic head model (comprising of scalp, skull, cerebrospinal fluid and brain) to allow modeling current flow distribution for rectangular sponge electrodes of different sizes and orientations.

## Results

### Effectiveness of blinding

After each session, the participants were asked to assess how certain they were about receiving stimulation or sham, dividing 100% between the two options (e.g., if someone was rather certain it was sham, the answer could be 80% sham, 20% stimulation). The certainty of a correct guess (i.e., certainty of receiving stimulation during the anodal tDCS or cathodal tDCS session and of receiving placebo in the sham session) varied between 45-57% (sham condition 45%, cathodal stimulation 55%, anodal stimulation 57%), indicating that our blinding procedure was successful.

### RTs and accuracy rates

Average RTs were calculated from correct responses for both target and non-target stimuli. For each participant, reaction times above or below 3 SD from the individual mean value were excluded as outliers (for justification of this approach, see Miller, 1991).

N-back accuracy was measured with sensitivity index (d-prime). To be able to calculate d-prime values even for extreme hit or false alarm rates of 0 or 1, the raw scores were transformed using the log-linear approach as suggested by Stanislaw and Todorov (1999).

Table 1 shows average RT and d-prime values in each block of each stimulation condition.

**Table 1:** RT (in milliseconds) and accuracy (log-linear d-prime) on the three stimulation conditions per block (Block 1 = pre-tDCS; Block 2 = during tDCS/online; Block 3 = after tDCS/offline). Mean (SD).

**Session**	**Anodal tDCS**			**Sham**			**Cathodal tDCS**		
Block	1	2	3	1	2	3	1	2	3
RT M	687	638	613	673	635	610	688	648	613
RT SD	175	149	144	196	185	169	172	159	138
d-prime M	2.94	2.92	3.15	3.01	3.03	3.21	2.78	2.76	3.07
d-prime SD	1.35	1.11	0.96	1.26	1.1	0.91	1.28	1.15	1.1

**Figure 2:**
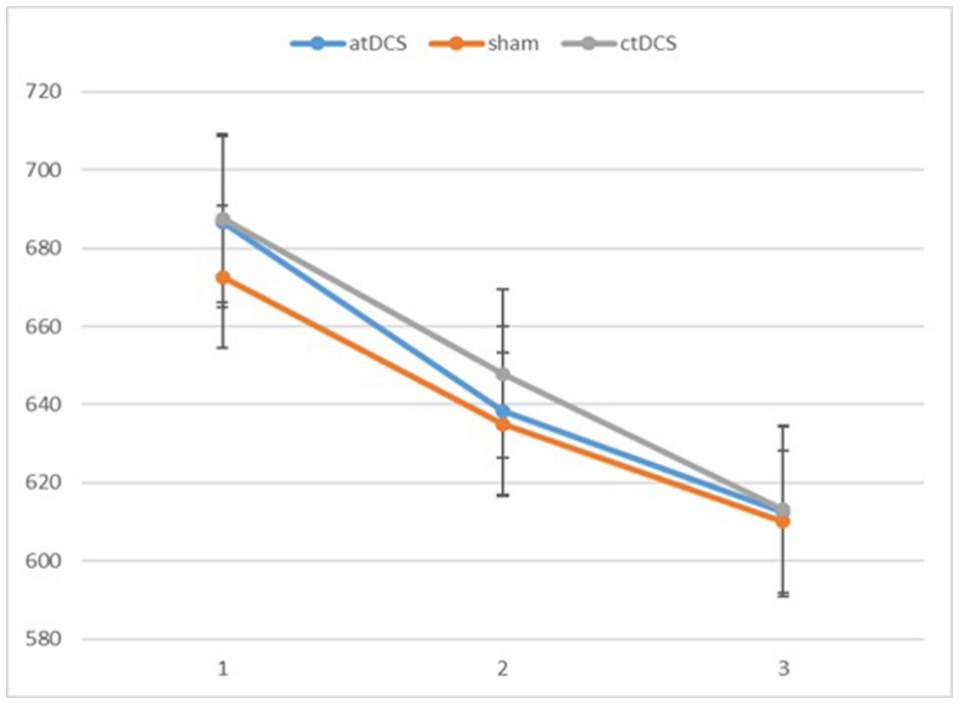
Participants' reaction times in the three blocks of each session (1 = baseline, 2 = online, 3 = offline). Error bars represent standard error.

**Figure 3:**
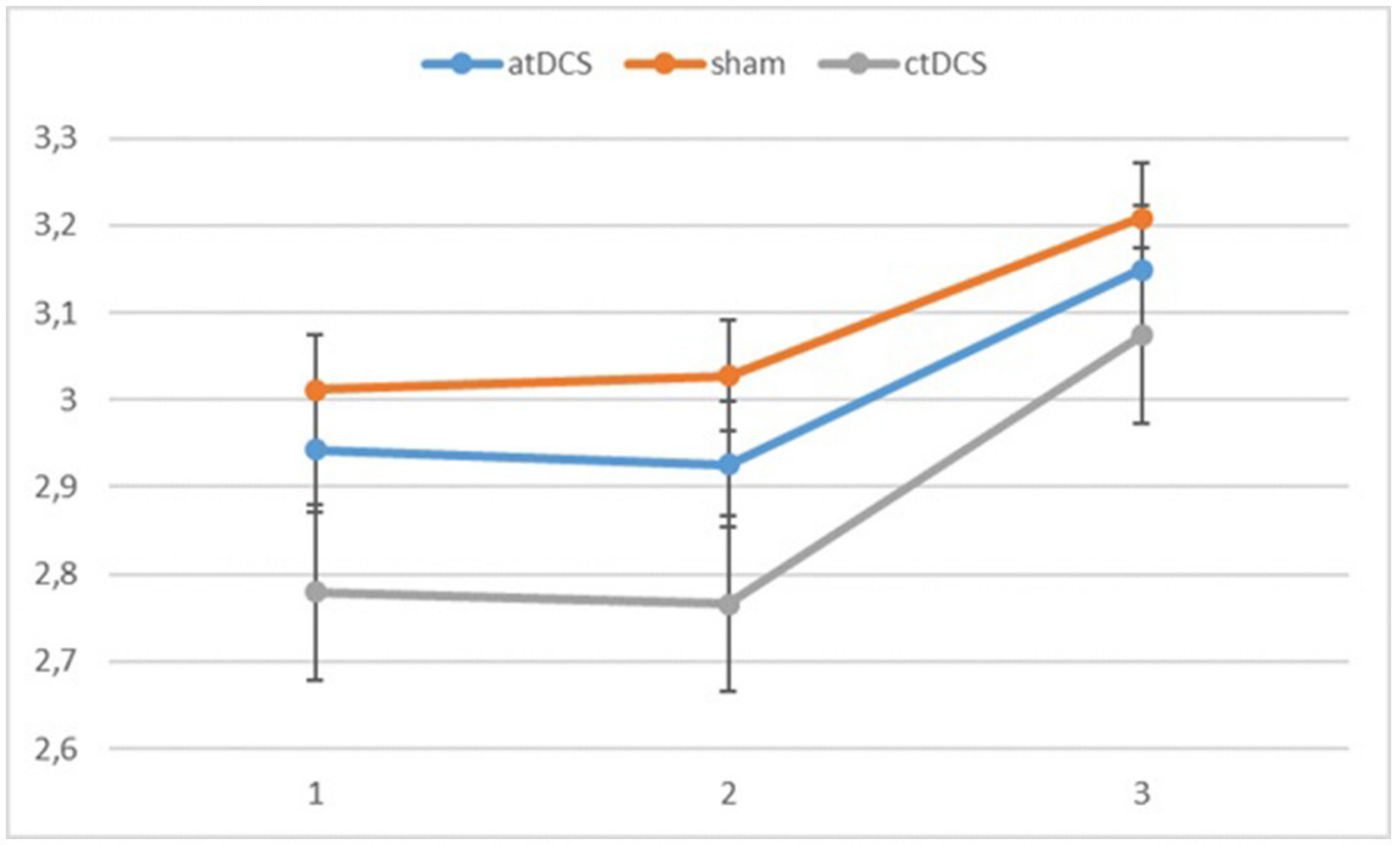
Log-linear d-prime values in the three blocks (1 = baseline, 2 = online, 3 = offline). Error bars represent standard error.

We conducted repeated-measures analyses of variance (ANOVAs) separately for RTs and d-primes. The results are reported in Figures 2, 3, respectively. For RTs, we observed a main effect of block, *F*_(2, 60)_ = 91.83, *p* < 0.001, ηp^2^ = 0.754, indicating that the participants became faster in the later blocks. The main effect of stimulation type was not significant, *F*_(2, 60)_ = 0.215, *p* = 0.807. There was no significant interaction between stimulation type and block, *F*_(4, 120)_ = 0.542, *p* = 0.706.

Also for the d-prime values, we observed a significant main effect of block, *F*_(2, 60)_ = 11.065, *p* < 0.001, ηp^2^ = 0.269 as the participants became more accurate throughout the sessions. Neither stimulation type, *F*_(2, 60)_ = 0.827, *p* = 0.442, nor its interaction with block, *F*_(4, 120)_ = 0.146, *p* = 0.964, were significant.

Overall, we did not observe significant effects of stimulation on RTs or accuracy rates. Main effect of block on both RTs and accuracy rates suggested a learning effect. Additional analyses were conducted to examine the role of individual variance in response to stimulation (gain-score analysis) and to separate between the stimulation effects for low- vs. high-performing participants.

### Gain score analysis

To analyze individual gains, which could be more informative than mean group scores, we calculated for each participant and session gain scores, that is, difference in performance between blocks. The gain scores were calculated as follows:

$$\begin{array}{l} Session\;X\;\left(block\;1\;performance\right)-Session\;X\;\left(block\;2\;performance\right)\\ Session\;X\;\left(block\;1\;performance\right)-Session\;X\;\left(block\;3\;performance\right) \end{array}$$

Thus, for each participant, in a given session (or stimulation type) we calculated two gain scores for both RTs and d-primes. We conducted a mixed-model ANOVA with stimulation type (3 levels) as a within-subjects factor, and session order as a between-subjects dummy variable, for each gain score separately. There was no significant effect of stimulation type in any of the analyses [RTs: *F*_(2, 60)_ = 0.770, *p* = 0.468 for gain in block 2, *F*_(2, 60)_ = 0.530, *p* = 0.591 for gain in block 3; d-primes: *F*_(2, 60)_ = 0.134, *p* = 0.875 for gain in block 2, *F*_(2, 60)_ = 0.041, *p* = 0.959 for gain in block 3]. Thus the participants' individual gains were not influenced by the stimulation condition.

### Median-split on baseline performance

As the first block of the first session can be considered as baseline for a participant's WM updating performance, we used performance level on that initial block to divide participants into groups of high-performers (*n* = 16 in RTs, *n* = 17 in accuracy) and low-performers (*n* = 17 in RTs, *n* = 16 in accuracy) based on median split. A series of independent samples *t*-tests revealed that for d-primes, the difference between groups was significant (*p* < 0.05) in all sessions and blocks except for second (*p* = 0.06) and third block (*p* = 0.1) in the anodal stimulation session. This is most likely a chance finding. On the other hand, for RTs, the difference between low- and high-performers was significant (*p* < 0.05) in all sessions and blocks of the task.

We conducted a mixed-model ANOVA for RTs and d-primes, with stimulation type and block as within-subjects factors and group (high- vs. low-performers) as a between-subjects factor, and session order as a between-subjects dummy variable.

In RTs, we observed a significant main effect of block, *F*_(2, 54)_ = 78.99, *p* < 0.001, ηp^2^ = 0.745, as RTs became faster overall over the blocks. The main effect of stimulation type was not significant, *F*_(2, 54)_ = 0.076, *p* = 0.927. The group × block interaction reached statistical significance, *F*_(2, 54)_ = 4.86, *p* = 0.01, ηp^2^ = 0.153. The low-performing group became faster consistently throughout the blocks, while for the high-performing group, the most notable gain took place in the second block. The block × stimulation type interaction was non-significant, *F*_(2, 54)_ = 0.33, *p* = 0.857. The three-way interaction was not significant either, *F*_(4, 108)_ = 0.481, *p* = 0.750.

**Figure 4:**
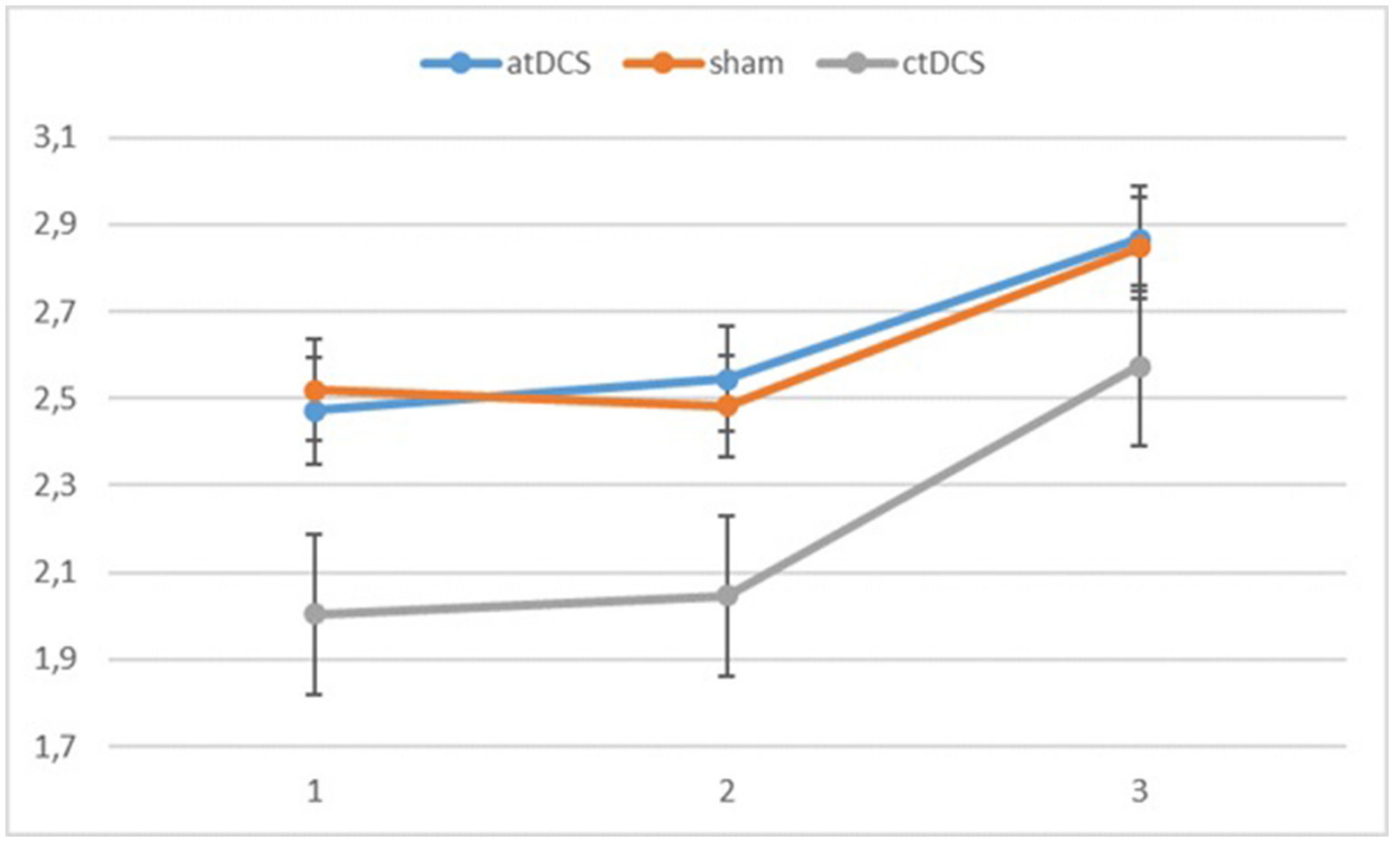
Changes in accuracy rates (d-prime) for the different stimulation types across blocks (1 = baseline, 2 = online, 3 = offline) in the low-performing group.

**Figure 5:**
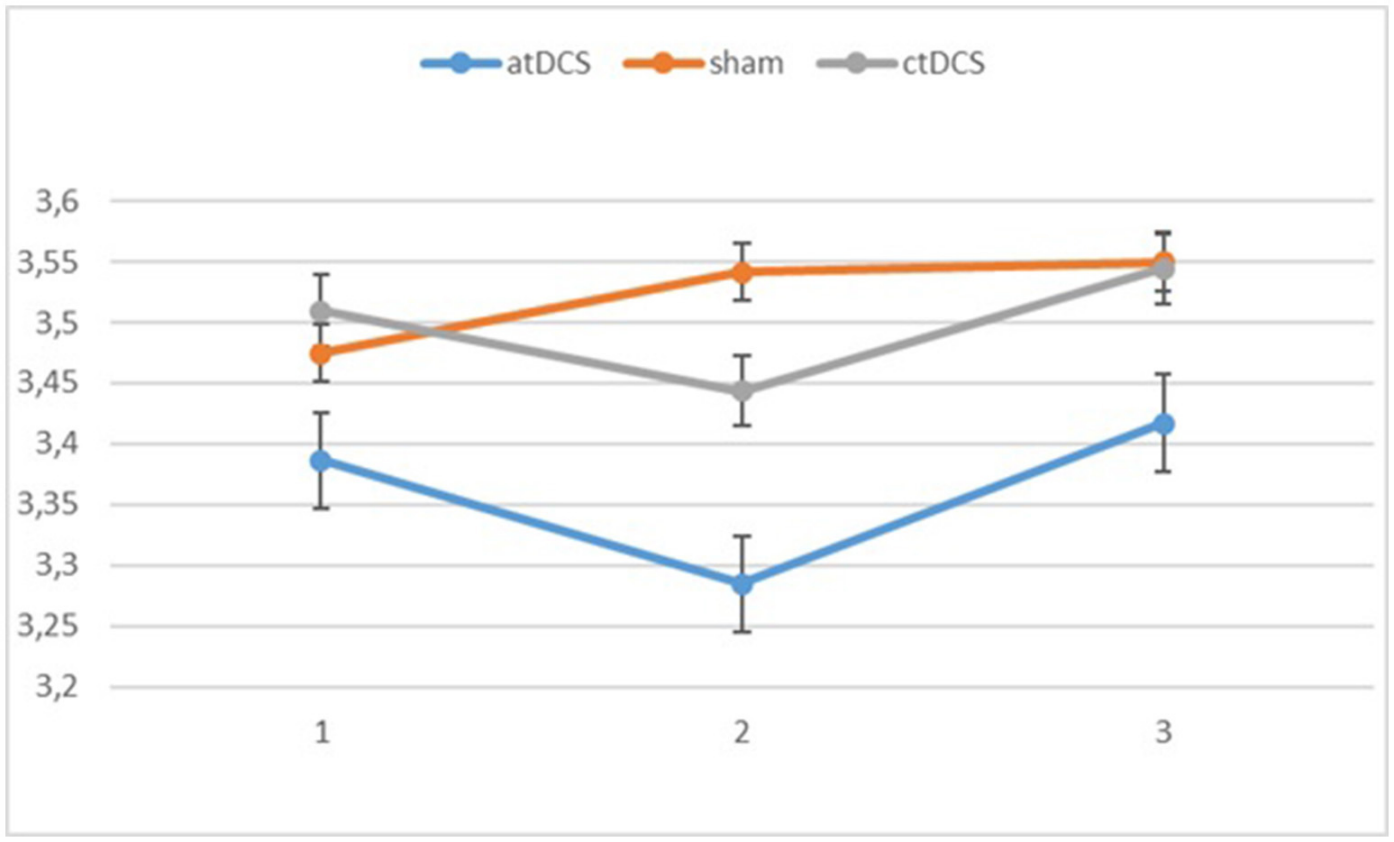
Changes in accuracy rates (d-prime) for different stimulation types across blocks (1 = baseline, 2 = online, 3 = offline) in the high-performing group.

Also with d-primes, we observed a significant main effect of block, *F*_(2, 54)_ = 16.362, *p* < 0.001, ηp^2^ = 0.377, with accuracy rates increasing over the sessions. The main effect of stimulation type was non-significant, *F*_(2, 54)_ = 1.02, *p* = 0.367. The group × block interaction was significant, *F*_(2, 54)_ = 8.68, *p* = 0.001, ηp^2^ = 0.243, stemming from a higher gain in accuracy in block 3 for the low-performing group. The group × stimulation interaction term was marginally significant, *F*_(2, 54)_ = 2.85, *p* = 0.066, ηp^2^ = 0.096. In the low-performing group (see Figure 4), stimulation had no effect on the rate of improvement in accuracy in the third block. However, in the high-performing group (see Figure 5), anodal and cathodal stimulation caused a slight deterioration of accuracy, which then recovered in the post-stimulation block to original or slightly higher levels. At the same time, the sham condition in this group showed a small increase of accuracy in the second block that was maintained at the same level in the third block. The block × stimulation type interaction was not significant, *F*_(4, 108)_ = 0.152, *p* = 0.962. The three-way interaction was not significant, *F*_(4, 108)_ = 0.352, *p* = 0.842.

### Electric current distribution modeling

**Figure 6:**
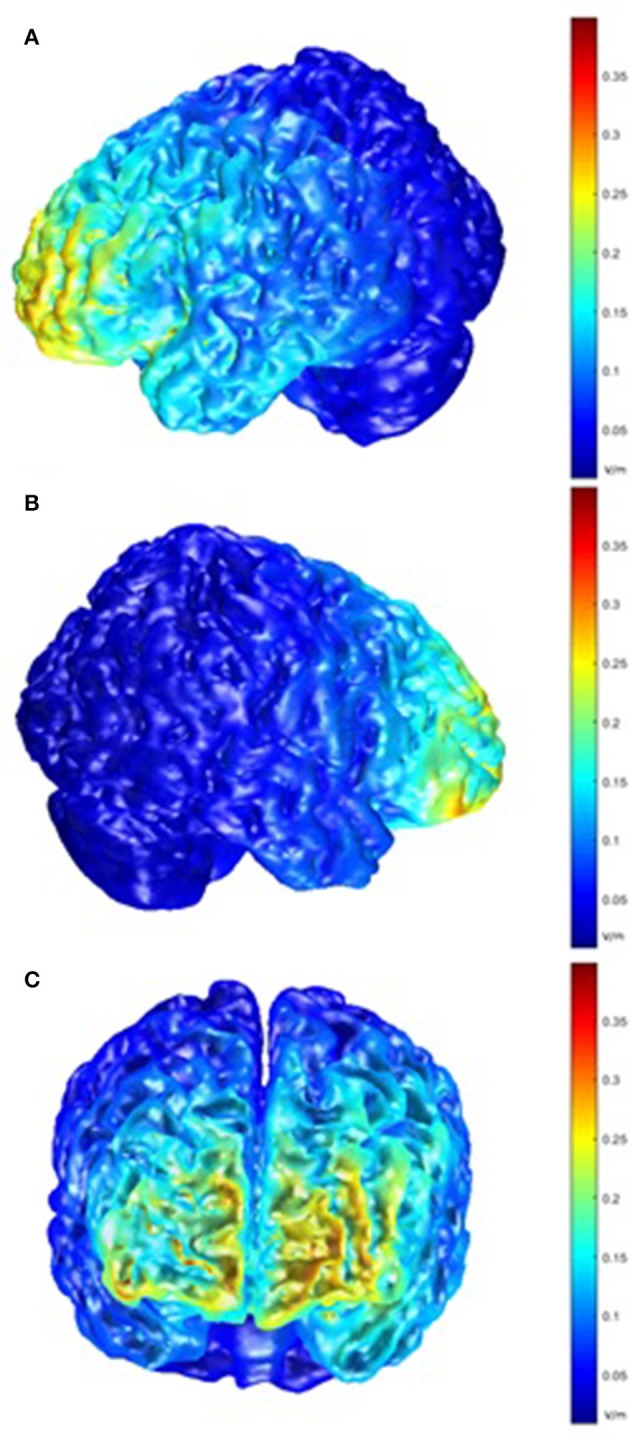
Model of electric field intensity (EFI; volts/meter) in anodal stimulation in the present montage. **(A)** left hemisphere view; **(B)** right hemisphere view; **(C)** frontal view.

Post-hoc modeling of the current flow for our montage was prompted by the lack of behavioral effects of the tDCS stimulation. For the left hemisphere, this modeling showed the highest electric field intensity (EFI) in the frontopolar cortex and the anterior parts of the left VLPFC and inferior frontal sulcus, extending to the DLPFC. On the right side, a slightly more circumscribed EFI pattern was seen, encompassing the frontopolar cortex and the anterior part of the right VLPFC (Figure 6).

## Discussion

The present study investigated the effects of anodal and cathodal tDCS on performance in a verbal 3-back task. We chose the left VLPFC as the target site for stimulation because, despite its key role in verbal WM processes, this region has not been targeted in previous tDCS studies relating to WM. In our double-blind cross-over design, all participants took part in three sessions that included either anodal, cathodal or sham stimulation. The three-block structure of each session allowed us to assess performance prior to, during (“online”), and after stimulation (“offline”). The participants improved on the n-back task over the course of each session. However, their n-back performance on the sham condition did not differ from either stimulation condition, either during stimulation or after it. To examine the role of inter-individual variability in stimulation effects and task performance, we conducted separate analyses with gain scores as the dependent variable, and also compared the stimulation effects for high- vs. low-performing participants. No statistically significant stimulation effects were observed in these analyses either.

Before discussing the potential reasons for the present results, it is worth noting the variability of findings in previous tDCS studies stimulating DLPFC during WM tasks. In their meta-analysis, Brunoni and Vanderhasselt (2014) found that tDCS to DLPFC enhanced participants' speed in the n-back task, but had no systematic influence on accuracy. Another meta-analysis by Hill et al. (2016) included a wide range of WM tasks. Their study reported that tDCS applied to DLPFC led to marginal improvement in both speed and accuracy, but only during the offline period following stimulation. However, the overall effects were small, and neither RT nor accuracy effects were consistent across the studies. The online or offline effects did not replicate from one study to another either (Hill et al., 2016). Approximately half of the studies targeting DLPFC have reported null effects or very small effects, suggesting that there are multiple factors that may affect the results. Therefore, the lack of stimulation effects in the present study are not entirely unexpected and future studies are needed to determine the reasons contributing to this variability in stimulation response across studies.

In our study, we observed no beneficial effect of tDCS on participants' reaction times in either the online or offline blocks. However, there was a statistically non-significant trend for lower accuracy during stimulation, but only in the high-performing group. This trend toward reduced accuracy was observed both with cathodal as well as anodal stimulation. Lack of polarity-specific effects are common in tDCS studies. For example, Wiethoff et al. (2014) showed no overall neurophysiological effect of cathodal tDCS administered to the motor cortex, and a substantial percentage of subjects in that study showed neural facilitation after both anodal and cathodal tDCS. Regarding cognitive function, a meta-analysis by Jacobson et al. (2012) concluded that the effects of cathodal stimulation are often not replicable.

One possible explanation for the lack of stimulation effects in our study could be the use of a relatively small (i.e., active) reference electrode, decreasing current density in the left hemisphere. Post hoc modeling of current flow for our montage demonstrated that portions of the right prefrontal cortex in the vicinity of the cathode were affected by stimulation. The use of a small electrode may have contributed to the observed electric field density in the right prefrontal cortex, and this may have diminished potential beneficial effects of anodal tDCS on the left VLPFC. Interestingly, the right ventral frontal cortex has been reported to be engaged in inhibition in the presence of unexpected events or stimuli (Aron et al., 2014), and possible cathodal tDCS effects might have interfered with this process. In fact, with more demanding WM tasks or with increased load within a task, bilateral frontal activation has been observed (Rypma and D'Esposito, 1999), which suggests that right frontal activation in WM tasks may be important for effective task performance. For example, in a verbal n-back study by Cohen et al. (1997), there was a more pronounced right frontal lobe activation during a demanding 3-back condition compared to a much less demanding 1-back condition, which the authors related to a greater involvement of the central executive system to balance the demands on both maintenance and updating. Since we employed a 3-back task, the involvement of the right frontal lobe could thus be expected. Our modeling also indicated that current flow was strongest in the most anterior parts of the left IFG, and more posterior regions (underneath the electrode) were not strongly affected. This may also have contributed to our null results, and the possible influence of electrode location should be studied further. Moreover, accompanying brain stimulation with objective electrophysiological measures, such as EEG or MEG, would shed light on how individual brain responsiveness interacts with tDCS, and would provide a valuable concomitant measure to behavioral tasks. Simultaneous brain activation measures were lacking in the present study.

It is also worth highlighting that we studied only a constant high WM updating load. Our decision to use the 3-back only was motivated by the relative difficulty of this condition; our previous research indicated that a 2-back task would be too easy for the university student sample, especially concerning the design with repeated sessions and inevitable learning effects, while 4-back would be too difficult. The focus on one condition allowed us to collect a large number of data points, which would not have been possible, had we chosen to manipulate the updating load. Further research is needed to study the possible effects of tDCS on systematically varied WM updating load.

Another possible explanation for the lack of stimulation effects is that VLPFC influences WM via different mechanisms than those of DLPFC. In DLPFC, higher activity is a reliable predictor of improved task performance (e.g., Braver et al., 1997). In turn, it has been shown that VLPFC, and IFG in particular, becomes activated in WM tasks in response to stimulus interference (Jonides and Nee, 2006). The IFG involvement in updating was more consistent for verbal material (Johnson et al., 2005). Zhang et al. (2003) propose that since information maintenance, interference resolution and updating converge in the left IFG in verbal tasks, it should be considered as the executive center for verbal WM. WM tDCS studies have not yet examined how stimulating the IFG affects those overlapping functions. The differences in WM-related functional roles exhibited by different brain regions call for studies that would directly compare different stimulation sites. As we stimulated only the VLPFC, we cannot infer here how this region differs from the more commonly studied DLPFC concerning responsiveness to tDCS during WM updating.

In addition, our results may have been affected by the use of a cross-over design in which all participants experienced two stimulation modes and placebo in a counter-balanced order. While the advantage of the cross-over design is the provision of within-subject baseline for stimulation effects, repeated task performance leads to learning and increased use of strategies that could mask possible stimulation effects. This may be particularly true for our highly specific subsample of healthy university students. Repeated exposure to the task allows more time to develop adaptive strategies. When spontaneous strategy use such as chunking and rehearsal in n-back is probed, it turns out to be very common even after a relatively short task exposure (Laine et al., 2017). Because implementation of strategies may result in substantial performance improvement, such effects may overshadow potentially smaller stimulation effects in counterbalanced cross-over designs. We did not probe our participants' strategy use, but some of them did spontaneously mention using strategies, and it is likely that many others were utilizing some strategy to complete the task. tDCS-induced changes are thought to be temporally dependent and thus it is suggested that one measures performance after the stimulation has ended, but there is no consensus as to the optimal time frame (see for example Ohn et al., 2008; Reis et al., 2013), and perhaps the posttest block in our study was too early to observe the after-effects of stimulation. However, even a single n-back session yields learning effects that can persist for weeks (Soveri et al., 2016). A potential remedy for tDCS studies using tasks that are prone to such effects could be to use between-subjects designs and carefully match participants on relevant demographic and baseline variables.

Our null findings could also reflect in part the substantial individual variation in response to tDCS. As previously mentioned, there might be subgroups that benefit more from tDCS or even show impaired performance. For example, Meinzer and colleagues demonstrated that tDCS over IFG improved performance on a word generation task in older participants with lower baseline performance and hyperactivity in right prefrontal regions (Meinzer et al., 2013). Such individual differences may be masked in group-level comparisons by averaging, and thus possible beneficial effects of stimulation may remain uncovered. Electrophysiological measures could help in targeting people who respond favorably to tDCS. While there were no significant effects in our groups of high vs. low performing young individuals, there was a non-significant trend toward impaired performance in the high performers during both anodal and cathodal stimulation. However, to date, there is little information about predictors of stimulation outcomes even in healthy populations and a number of factors may interact with state or trait variables in individuals (Fertonani and Miniussi, 2017). For example, Kim et al. (2014) studied the relationship between performance on a verbal 3-back task and tDCS current density modeled on the basis of individual magnetic resonance imaging data. They concluded that participants showing enhanced WM performance after tDCS also had a significantly larger current density at the stimulation site compared to low-performing participants. This suggests that, for tDCS to improve performance, the current at the stimulation site should reach certain levels, which depends on numerous physiological and anatomical factors. Modeling studies have reported that bone thickness, subcutaneous fat levels, cerebrospinal fluid density and cortical surface topography have an impact on current flow and density during stimulation (Datta et al., 2012; Truong et al., 2013). Some studies suggest that even personality traits may affect responses to tDCS (Peña-Gómez et al., 2011). These findings bring up questions as to who would gain most from brain stimulation and give some clues for future directions of tDCS research.

## Conclusions

The present study investigated the effects of tDCS applied to the left VLPFC on verbal WM updating during the task (online) and afterwards (offline) in a sample of young, healthy university students. We observed no effects of the stimulation on 3-back task performance in either RTs or accuracy measures. Based on previous tDCS studies, there is a number of factors that may explain the obtained null results. We therefore suggest several future directions for tDCS studies targeting the left VLPFC during or after WM task performance. Group-level comparisons may have masked some individual differences in responsiveness to tDCS, as we observed a trend toward performance decrease after stimulation in high-performing participants. More research is needed to explore the factors causing inter-individual variability in responsiveness to tDCS. It is also possible that tDCS effects could not be observed immediately after the stimulation, and that the consolidation effects induced by the stimulation would be observed only at longer time intervals. Our findings contribute to the exploration of effects of non-invasive brain stimulation in a healthy population and should help in the re-examination of the existing stimulation protocols for most promising results.

## Ethics statement

The study was approved by the Ethics Committee for The Hospital District of Southwest Finland. All participants gave a written informed consent prior to participation. They were briefed upon arriving in the lab and informed that they can withdraw from the study at any point.

## Author contributions

All authors contributed extensively to the work presented in this paper. MM, MaL, MiL, JS, and KL designed the experiment. MiL, JS, and KL administered the experiment, collected data and analyzed it. JJ conducted the medical screening of participants. MM and KL carried out the current distribution modeling. KL wrote the manuscript. All authors discussed the results and implications and commented on the manuscript at all stages.

### Conflict of interest statement

The authors declare that the research was conducted in the absence of any commercial or financial relationships that could be construed as a potential conflict of interest.
